# Modifiable cardiovascular risk factors in adults aged 40–79 years in Germany with and without prior coronary heart disease or stroke

**DOI:** 10.1186/s12889-015-1929-5

**Published:** 2015-07-24

**Authors:** Julia Truthmann, Markus A. Busch, Christa Scheidt-Nave, Gert B. M. Mensink, Antje Gößwald, Matthias Endres, Hannelore Neuhauser

**Affiliations:** Department of Epidemiology and Health Monitoring, Robert Koch Institute, Berlin, Germany; German Center for Cardiovascular Research (DZHK), Berlin, Germany; Department and Out-Patient Care of Neurology, Charité, Universitätsmedizin Berlin, Berlin, Germany; Center for Stroke Research Charité, Universitätsmedizin Berlin, Berlin, Germany; German Center for Neurodegenerative Diseases (DZNE), Berlin, Germany

**Keywords:** Coronary heart disease, Stroke, Prevention, Risk factors

## Abstract

**Background:**

Control of modifiable cardiovascular disease (CVD) risk factors has substantially reduced CVD mortality, but risk factor levels in populations may change and need continuous monitoring. This study aims to provide current estimates of the prevalence of these risk factors in Germany according to sex and history of coronary heart disease (CHD) or stroke.

**Methods:**

The analyses were based on data from the German Health Interview and Examination Survey for Adults (DEGS1; age 40–79 years, *n* = 5101), which is a cross-sectional population-based examination survey. CVD risk factors were defined according to recommendations in the European Guidelines on Cardiovascular Disease Prevention in Clinical Practice 2012.

**Results:**

The mean age was 57 years and 52 % were female; 493 participants had prior CHD and 163 participants a prior stroke. The overall prevalence of behavioural risk factors ranged from 17.9 % for high risk alcohol consumption to 90 % for low vegetable intake. Blood pressure ≥ 140/90 mmHg was found in 21 % and 69 % had total cholesterol ≥ 5.0 mmol/l. Only 16 % met the targets for five behavioural factors combined (smoking, physical activity, fruit intake, alcohol intake and obesity), 13 % of those with and 16 % of those without CHD or stroke. The prevalences of most behavioural risk factors were higher among men compared to women.

**Conclusions:**

There is a high prevention potential from modifiable cardiovascular risk factors in the general population aged 40–79 years in Germany and among those with prior CHD or stroke. Risk factors are often co-occurring, are interrelated and require combined educational, behavioral, medical and policy approaches.

**Electronic supplementary material:**

The online version of this article (doi:10.1186/s12889-015-1929-5) contains supplementary material, which is available to authorized users.

## Background

Cardiovascular disease (CVD) is the most common cause of death before the age of 65 in Europe [1]. However, a substantial proportion of these deaths may be preventable through changes in lifestyle [2]. The European Guidelines on cardiovascular disease prevention in clinical practice 2012 (ESC 2012) [3] made the following key recommendations to lower CVD risk: quit smoking, increase physical activity, eat a healthy diet, limit alcohol consumption, reduce body weight, reduce blood pressure (BP) and control blood lipids. Men and women with established CVD are at very high risk for future events and prompt interventions on risk factors are recommended. However, CVD risk factors often remain uncontrolled [4, 5], and up-to-date data on the prevention potential of modifiable risk factors in the general population as well as in high-risk groups are difficult to obtain.

The aim of this cross-sectional study is to contribute to the debate on CVD prevention by providing up-to-date estimates of CVD risk factor proportions of the principal modifiable CVD risk factors in adults aged 40–79 years in Germany with data from the nationwide population-based German Health Interview and Examination Survey for Adults (DEGS1) [6]. In addition, the study presents risk factor prevalences among persons with and without prior coronary heart disease (CHD) or stroke.

## Methods

### Study design and sample

**Fig. 1:**
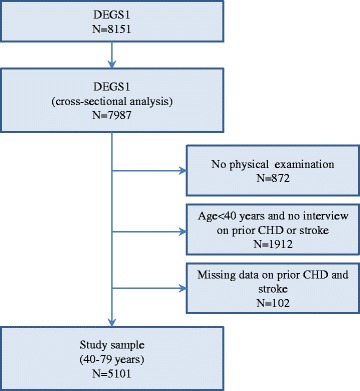
Exclusion criteria. CHD: Coronary heart disease

The first wave of the German Health Interview and Examination Survey for Adults (DEGS1) was conducted in 2008–2011 with the aim to obtain comprehensive information about the health of the residential population aged 18–79 years living in Germany. Design and methods were described in detail elsewhere [6–8]. The survey comprises 8151 men and women who live in Germany, including 4192 first-time participants and 3959 persons who already participated in the German National Health Interview and Examination Survey 1998 (GNHIES98) [8]. Exclusion criteria for the present analysis are presented in Fig. 1 resulting in a final study sample of 5101 participants (2436 men, 2665 women).

The DEGS1 study was approved by the Federal and State Commissioners for Data Protection and the Charité - Universitätsmedizin Berlin ethics committee (No. EA2/047/08). All participants provided written informed consent prior to the interview and examination.

### Data collection and study variables

The modifiable risk factors were defined according to recommendations of the ESC 2012 guideline [3]. As part of a general self-administered health questionnaire, daily smoking was assessed with several questions. Physical activity within a usual week was also assessed within this questionnaire and defined as low when < 2.5 h physical activity/week [9] and physical inactive when no physical activity was reported. A semi-quantitative self-administered food frequency questionnaire was used to assess the frequency and portion size of 53 food items for a reference period of the past four weeks. The number of servings of fruit (fresh and cooked) and vegetable (fresh and cooked vegetable, legumes) and servings of fish (cold fish and fish as a hot meal) per day were calculated by combining the frequency of consumption and the usual portion size. Low fruit intake was defined as < 2 portions fruit per day, low vegetable intake as < 2 portions vegetable per day and low fish intake as < 1 portion fish per week. Quantity of alcohol intake was calculated by summing the alcohol content of consumed beer, non-alcoholic beer (still containing minute alcohol amounts), wine, spirits [10] and cocktails (assuming one cocktail to contain 15.0 g of alcohol [11]). High alcohol consumption was defined as > 10 g alcohol per day in women and > 20 g alcohol per day in men. Since zero alcohol consumption has been related to increased cardiovascular risk and mortality, a separate category was defined as alcohol intake of 0 g/day [3]. Body weight was measured with an electronic scale and body height was measured using a portable stadiometer. The body mass index (BMI) was calculated as BMI [kg/m^2^] = body weight [kg]/body height^2^ [m]. A BMI ≥ 25 kg/m^2^ was defined as overweight and a BMI ≥ 30 kg/m^2^ was defined as obesity [12]. Three sitting blood pressure (BP) measurements were taken after a five-minute rest on the right arm following a standardized protocol with an automated oscillometric device (Datascope Accutorr Plus) and the prevalence of BP above ≥ 140 mmHg systolic or above ≥ 90 mmHg diastolic was determined using the mean of the second and third BP measurements. Total cholesterol (TC) and high density lipoprotein cholesterol (HDL) were measured using an enzymatic assay (Architect ci8200, Abbott, Germany). Elevated TC was defined as a value ≥ 5.0 mmol/l (190 mg/dl) and an additional cut-off of 6.2 mmol/l (240 mg/dl) was included based on previous guidelines [13]. Non-HDL was calculated by subtracting HDL from TC. Elevated non-HDL was defined as ≥ 3.8 mmol/l among participants without prior CHD or stroke and ≥ 2.6 mmol/l among those with prior CHD or stroke. Previous diagnoses of CHD, stroke, hypertension and dyslipidaemia were assessed in a standardized physician interview. Medications taken in the previous seven days were coded according to the Anatomical Therapeutic Chemical (ATC) classification system. Medications from ATC-group C10 were counted as lipid-lowering when dyslipidaemia was known. Similarly, medications from ATC groups C03, C07, C08, C09 and C02 were defined as antihypertensive in those with known hypertension.

### Analysis

For the descriptive analyses percentages and 95 % confidence intervals were calculated. In order to account for the unequal sampling probabilities related to the sampling design and nonresponse, statistical analyses were weighted using a weighting factor [8] and the complex survey sampling was taken into account using complex samples procedures in SPSS version 20.0 (SPSS Inc., Chicago, Illinois, USA). P values less than 0.05 were considered statistically significant. The analyses were stratified for prior CHD or stroke, sex and age. Furthermore, the prevalences of cardiovascular risk factors were calculated according to obesity status, BP level and TC level group. For analysis of behavioural risk factor combinations, we considered five categories represented by one indicator each: daily smoking, physical activity <2.5 h/week, < 2 portions of fruit/day, alcohol intake >10 g/day in women or >20 g/day in men, and BMI ≥ 30 kg/m^2^.

## Results

The mean age of the study population was 57 years and 52 % were female. The prevalence of CHD was 9 % (95 % CI 8–10) and the prevalence of stroke was 3 % (95 % CI 2–3). The prevalence of prior CHD or stroke was 11 % (95 % CI 10–12). The mean age of the group with CHD or stroke was 67 years and 35 % were female. Table 1 shows the distribution of cardiovascular risk factors for the overall sample and for participants with and without prior CHD or stroke separately. The overall prevalence of behavioral risk factors ranged from 18 % for high risk alcohol consumption and 20 % for daily smoking to 82 % for low physical activity and 90 % for low vegetable intake. BP ≥ 140/90 mmHg was found in 21 % and 69 % had TC ≥ 5.0 mmol/l (including 27 % with TC ≥ 6.2 mmol/l).

**Table 1:** Prevalence of cardiovascular risk factors (95 % confidence interval) among adults aged 40–79 years

Risk factor	Total (*N* = 5101)	No CHD or stroke (*N* = 4489)	CHD or stroke (*N* = 612)	P
Daily smoking	19.8 (18–21.7)	20.2 (18.3–22.2)	16.5 (12.4–21.6)	0.200
<2.5 h physical activity/week	82.4 (80.9–83.7)	82.2 (80.7–83.6)	84.1 (79.9–87.6)	0.400
Physically inactive (no physical activity in average week)	34.2 (32.3–36.4)	32.4 (30.3–34.6)	49.8 (44.4–55.3)	**<0.001**
<2 portions fruit/day	66.1 (64.3–67.8)	65.8 (63.9–67.7)	68.1 (63–72.8)	0.400
<2 portions vegetable/day	90.4 (89.2–91.5)	90.1 (88.8–91.3)	92.8 (89.3–95.2)	0.100
<1 portion fish/week	67.3 (65.5–69.1)	66.8 (64.9–68.7)	71.6 (66.5–76.2)	0.100
Alcohol intake > 10 g/day (women) or 20 g/day (men)	17.9 (16.5–19.4)	18 (16.7–19.5)	16.7 (12.9–21.4)	0.600
Alcohol intake = 0 g/day	12.7 (11.3–14.3)	12.4 (10.8–14.2)	15.2 (11.7–19.5)	0.200
Overweight (BMI ≥ 25 kg/m^2^)	69.9 (68.2–71.5)	68 (66.3–69.8)	85.1 (81.2–88.3)	**<0.001**
Obesity (BMI ≥ 30 kg/m^2^)	28.4 (26.6–30.2)	26.5 (24.8–28.3)	43.1 (37.4–48.9)	**<0.001**
BP ≥ 140/90 mmHg	20.6 (18.9–22.4)	20.7 (18.9–22.5)	20 (16.1–24.5)	0.700
Proportion unaware^a^ among those with BP ≥ 140/90 mmHg	33.5 (30.2–37)	36 (32.5–39.7)	12.1 (6.6–21.3)	**<0.001**
Proportion treated and aware^b^ among those with BP ≥ 140/90 mmHg	45.4 (41.9–49)	42.5 (38.8–46.3)	70.6 (59.2–79.9)	**<0.001**
Elevated TC (≥5.0 mmol/l)	68.8 (66.5–71)	71.2 (68.9–73.5)	48.9 (43.6–54.1)	**<0.001**
Highly elevated TC (≥6.2 mmol/l)	24.7 (22.9–26.6)	25.9 (23.9–27.9)	15.2 (11.6–19.7)	**<0.001**
Proportion unaware^a^ among those with TC ≥ 5.0 mmol/l	57.5 (55.3–59.7)	59.4 (57–61.7)	35.0 (28.6–42)	**<0.001**
Proportion treated and aware^b^ among those with TC ≥ 5.0 mmol/l	9.1 (8–10.3)	7.5 (6.4–8.7)	28.3 (22.4–35.1)	**<0.001**

P values less than 0.05 were considered statistically significant (bold)

*BMI* body mass index, *BP* blood pressure, *CHD* coronary heart disease, *TC* total cholesterol

^a^defined as reported diagnosis of hypertension or dyslipidaemia

^b^defined as reported diagnosis of hypertension or dyslipidaemia in combination with reported use of antihypertensive or lipid-lowering drugs, respectively

The prevalence of behavioural risk factors (daily smoking, insufficient physical activity, insufficient fruit, vegetable and fish intake, high alcohol intake) was similar in participants with and without prior CHD or stroke. Participants with CHD or stroke were significantly more likely to be physically inactive, overweight and/or obese. Prevalence of BP ≥ 140/90 mmHg was similar in both groups. Participants with prior CHD or stroke were less likely to have elevated TC, but had a higher prevalence of elevated non-HDL (≥2.6 mmol/l; see Additional file 1, Table S1) compared to participants without prior CHD or stroke (≥3.8 mmol/l). Also, participants with prior CHD or stroke with BP ≥ 140/90 mmHg or elevated TC were less likely to be unaware of their hypertension or hyperlipidaemia, respectively and those aware were more likely to be treated.

The proportions of risk factors stratified for men and women are shown in Table 2. Compared to women, the prevalence of smoking, low fruit, vegetable and fish intake, high alcohol intake, overweight, BP ≥ 140/90 mmHg and non-HDL ≥ target was higher among men. In contrast, women were more often physically inactive and exceeded more often both cut offs for TC.

**Table 2:** Prevalence of cardiovascular risk factors (95 % confidence interval) among men and women aged 40–79 years

Risk factor	Men				Women			
	Total (*N* = 2436)	No CHD or stroke (*N* = 2040)	CHD or Stroke (*N* = 396)	P	Total (*N* = 2665)	No CHD or stroke (*N* = 2449)	CHD or Stroke (*N* = 216)	P
Daily smoking	21.6	22.4	16.4	0.100	18	18.2	16.7	0.700
	(19.1–24.3)	(19.5–25.6)	(11.5–22.9)		(16.1–20.2)	(16.2–20.3)	(10.2–26)	
<2.5 h physical activity/week	80	79.6	82.7	0.300	84.7	84.5	86.4	0.600
	(77.8–82.1)	(77.2–81.8)	(76.9–87.3)		(82.8–86.4)	(82.5–86.3)	(79–91.5)	
Physically inactive (no physical activity in average week)	32.3	29.8	48.1	**<0.001**	36.2	34.8	52.9	**<0.001**
	(29.7–35.1)	(27–32.7)	(40.8–55.5)		(33.6–39)	(32.1–37.7)	(44–61.7)	
<2 portions fruit/day	71	71.5	67.7	0.300	61.3	60.7	68.8	0.100
	(68.5–73.3)	(68.9–74)	(60.9–73.9)		(58.9–63.7)	(58.2–63.1)	(60.8–75.8)	
<2 portions vegetable/day	93.9	93.8	93.7	0.900	87	86.7	90.7	0.200
	(92.4–95.1)	(92.7–95.1)	(88.8–96.9)		(85–88.8)	(84.5–88.6)	(85.1–94.3)	
<1 portions fish/week	66.5	64.8	70.2	0.100	69.1	68.6	74	0.200
	(63.1–67.9)	(62.2–67.3)	(64.1–75.6)		(66.5–71.5)	(66–71.2)	(65.7–80.8)	
Alcohol intake > 10 g/day (women) or 20 g/day (men)	21.4	21.3	21.9	0.800	14.4	15	7.6	0.100
	(19.2–23.6)	(19.1–23.6)	(16.5–28.5)		(12.7–16.3)	(13.2–16.9)	(3.5–15.6)	
Alcohol intake = 0 g/day	8	8	8.5	0.800	17.4	16.6	27.1	**0.005**
	(6.6–9.6)	(6.3–10)	(5.1–13.7)		(15.3–19.8)	(14.4–19.2)	(20.2–35.3)	
Overweight (BMI ≥ 25 kg/m^2^)	77.6	76.2	85.9	**0.001**	62.4	60.5	83.6	**<0.001**
	(75.4–79.6)	(73.8–78.5)	(81.1–89.7)		(60–64.8)	(58–63)	(77.2–88.5)	
Obesity (BMI ≥ 30 kg/m^2^)	27.8	25.7	40.3	**<0.001**	28.9	27.2	47.8	**<0.001**
	(25.2–30.6)	(23.2–28.5)	(33.2–47.8)		(26.5–31.4)	(24.8–29.8)	(38.8–57)	
BP ≥ 140/90 mmHg	23.4	23.8	20.9	0.300	17.8	17.8	18.4	0.800
	(21.1–25.9)	(21.4–26.4)	(16.1–26.7)		(15.8–20.1)	(15.7–20.1)	(12.4–26.2)	
Proportion of unaware^a^ among those with BP ≥ 140/90 mmHg	36.1	39.6	10.3	**<0.001**	30.3	31.6	15.6	0.100
	(31.3–41.1)	(34.6–44.9)	(4.2–23.1)		(25.5–35.5)	(26.5–37.2)	(6.7–32.3)	
Proportion treated and aware^b^ among those with BP ≥ 140/90 mmHg	40.5	36	73.2	**<0.001**	51.7	50.4	65.6	0.100
	(35.3–46.1)	(30.6–41.8)	(58–84.3)		(46.6–56.8)	(45.3–55.6)	(46.9–80.5)	
Elevated total cholesterol (TC ≥ 5.0 mmol/l)	65.2	68.8	43.6	**<0.001**	72.3	73.4	59.4	**<0.001**
	(62.1–68.2)	(65.6–71.9)	(33.6–49.5)		(69.7–74.8)	(70.7–76)	(50.9–67.3)	
Highly elevated TC (≥6.2 mmol/l)	22.8	24.5	12.6	**0.001**	26.5	27.1	19.7	0.100
	(20.4–25.4)	(21.9–27.3)	(8.3–18.6)		(24.1–29.1)	(24.6–29.8)	(13.6–27.8)	
Proportion of unaware^a^ among those with TC ≥ 5.0 mmol/l	57.3	59.4	36.5	**<0.001**	57.7	59.4	33.1	**<0.001**
	(53.9–60.6)	(55.7–62.9)	(27.2–47.1)		(54.7–60.7)	(56.3–62.5)	(23.6–44.2)	
Proportion treated and aware^b^ among those with TC ≥ 5.0 mmol/l	8.9	6.8	29.7	**<0.001**	9.3	8.1	26.6	**<0.001**
	(7.3–10.7)	(5.4–8.4)	(21.1–40.1)		(7.7–11.2)	(6.5–10)	(18.5–36.7)	

P values less than 0.05 were considered statistically significant (bold)

*BMI* body mass index, *BP* blood pressure, *CHD* coronary heart disease, *TC* total cholesterol

^a^defined as reported diagnosis of hypertension or dyslipidaemia

^b^defined as reported diagnosis of hypertension or dyslipidaemia in combination with reported use of antihypertensive or lipid-lowering drugs, respectively

Age-group specific analyses revealed higher or equal proportions of most unfavourable risk factor levels in the older age group (see Additional file 1). As exceptions, smoking prevalence and proportion of unaware persons with elevated TC (≥5.0 mmol/l) were lower in older men and women and high alcohol intake was less frequent in older women.

The risk factor prevalences stratified by obesity group, BP target and TC target groups are presented in Additional file 2. Compared to non-obese participants, prevalence of BP ≥ 140/90 mmHg and zero alcohol consumption was higher among obese participants. In contrast, non-obese participants were more often physically inactive and exceeded more often the recommended alcohol intake. Compared to participants with BP < 140/90 mmHg prevalence of alcohol intake, TC ≥ 5.0 mmol/l, overweight and obesity was higher, and the prevalence of smoking was lower among the group with BP ≥ 140/90 mmHg. Except for BP, no difference could be observed between the TC target groups.

**Fig. 2:**
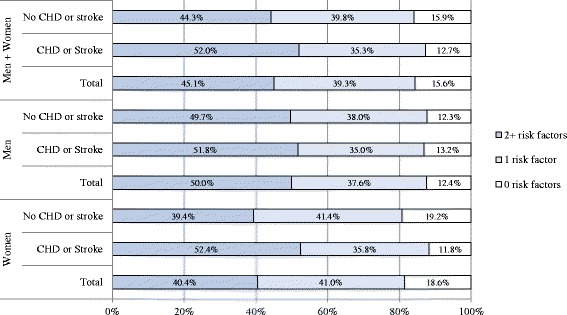
Combination of five behavioural risk factors (%) in adults aged 40–79 years with and without coronary heart disease (CHD) and stroke. The prevalences were stratified for sex and group with and without coronary heart disease (CHD) and stroke. The following behavioural risk factor categories were included: daily smoking, physical activity <2.5 h/week, < 2 portions of fruit/day, alcohol intake >10 g/day in women or >20 g/day in men, BMI ≥ 30 kg/m^2^

Figure 2 shows the combination of five behavioural risk factors. Only 15 % had none of these risk factors, 39 % had one risk factor, and 45 % had two or more risk factors. More women than men had no risk factor (12 % (95 % CI 11–14) vs. 19 % (95 % CI 17–21)). More than half of the CHD or stroke group and about 44 % of the group without prior CHD or stroke had two or more risk factors. Women with CHD or stroke more often had a combination of two or more behavioural risk factors compared to women without CHD or stroke (52 % (95 % CI 44-61) vs. 39 % (95 % CI 37-42)).

## Discussion

The results of this cross-sectional analysis of modifiable cardiovascular risk factors in adults in Germany suggest that, for the vast majority of adults in Germany, there is a high prevention potential for all of the behavioural risk factors investigated in this study: daily smoking, insufficient fruit, vegetable and fish consumption, high alcohol intake, insufficient physical activity and overweight. Almost 85 % of adults aged 40–79 years have at least one of these risk factors, with 45 % having a combination of two or more risk factors.

Studies on CVD risk factors in different countries are often difficult to compare due to differences in the specific combinations and definitions of the risk factors that were assessed. In the United States adherence to five healthy behavioural factors (including fruit and vegetable consumption, regular exercise, healthy weight, alcohol consumption and smoking) in 40 to 74 year olds was as low as 8 % in 2006 and had decreased from 15 % in 1988 [14]. A recent analysis from Italy on six cardiovascular risk factors (smoking, low fruit/vegetable consumption, obesity/overweight, hypertension, dyslipidaemia and diabetes) found that 90 % of the study population had more than one and 84 % had between two and five risk factors [15].

For BP and TC, we report prevalences of elevated, i.e., uncontrolled values as measured in our study. These prevalences do not include controlled hypertension or controlled hypercholesterolemia. It is encouraging that the target BP (<140 mmHg systolic and < 90 mmHg diastolic) was reached by almost 80 % of the participants but this proportion may be lower among severely ill patient groups. For comparison, in the SHARE survey the more stringent targets of < 140/90 mmHg in general and < 130/80 mmHg for patients with co-morbidities or high CV risk were reached by less than 60 % of general practice patients and by less than half of patients treated by cardiologists/internists [16]. The joint ESC 2012 guideline targets for hyperlipidaemia are mainly based on low-density lipoprotein (LDL) levels. We used TC and non-HDL since fasting level LDL data were not available for a sufficient number of participants. However, TC and non-HDL are established markers for increased cardiovascular risk and are independent of fasting time [17, 18]. The TC targets were more often reached by the CHD or stroke group than by the group without CHD or stroke, which is similar to previous findings [19] and most likely reflects more intensive lipid-lowering therapy among people with prior CHD or stroke. However, almost 90 % of the CHD or stroke group exceeded the stringent non-HDL target.

Except for physical inactivity, prevalence of behavioural risk factors was higher among men. Other studies found similar results concerning physical activity [20], smoking [5, 21], BMI [15] and fruit and vegetable intake [15]. Possible reasons include gender-specific cultural norms relating to lifestyle [22] and gender-specific responses to lifestyle. Higher prevalences for most modifiable risk factors were found among the age group 60–79 years compared to the younger group. This indicates the need for preventive interventions at early life stages, because the risk of CVD increases with the duration of risk factor exposure [3].

The strengths of our study include the large nationwide sample of community-dwelling adults, the highly standardized measurements and the detailed questionnaires. The most important limitation is that severely ill and institutionalized men and women were mainly not included in the study which may have led to an underestimation of the risk factor proportions and to the rather low stroke and CHD prevalence. Equally important, the group without prior CHD or stroke includes individuals in all risk categories including high risk. It is a heterogeneous group which is not in the main focus of the paper. Therefore, the main finding is that uncontrolled risk factors are currently still highly prevalent both in the overall group (general population) and in the group with prior CHD or stroke, a clearly high-risk group likely to be aware of this high risk. A further limitation is the self-reported diagnosis of hypertension and dyslipidaemia which may have led to an underestimation of the prevalence of these diseases. However, information on prior diagnosis was used only to calculate awareness of elevated BP and elevated TC.

The risk factors in this analysis are all among the top risk factors contributing to the Global Burden of Disease both 1990 and 2010 [23]. It is never too late for improvements in behavioural risk factors, as illustrated by a 35 % reduction of CVD events in 4 years in adults aged 45 years and older who switched to a healthy lifestyle [2]. Clearly, to individuals and to treating physicians it may appear more intuitive to put effort into lifestyle changes and enrol in prevention interventions in high-risk situations [24] where the number-needed-to-treat to prevent an event is low. However, population benefits are larger if healthy lifestyles are adopted early on in order to prevent or delay hypertension, dyslipidaemia, obesity and chronic diseases [25, 26]. Action is required both at the health care system and societal level. Individuals may need treatment, but more often they need knowledge, motivation and accessible, convenient and attractive opportunities for improved lifestyle. For example, health education through mass media has been shown to be cost-effective to limit CVD [27]. The key to population-wide risk factor changes is clearly a combination of personal and non-personal interventions.

## Conclusion

In conclusion, our study suggests that there is still a high prevention potential for adults in the general population and in the group with prior CHD or stroke in Germany and that prevention programs should aim at several major risk factors and their interrelations.

## Additional files

Additional file 1:
**Non-HDL target and prevalence of cardiovascular risk factors stratified for age group (40–59, 60–79).**
Additional file 2:
**Prevalence of cardiovascular risk factors by obesity status, blood pressure level, and total cholesterol level.**

## Abbreviations

ATC: Anatomical therapeutic chemical
BMI: Body mass index
BP: Blood pressure
CHD: Coronary heart disease
CVD: Cardiovascular disease
LDL: Low-density lipoprotein
TC: Total cholesterol
