# Supplementary material for: Modifiable cardiovascular risk factors in adults aged 40–79 years in Germany with and without prior coronary heart disease or stroke
Source: BMC Public Health. 2015 Jul 24;15:701. doi: 10.1186/s12889-015-1929-5 (PMC4513378; doi:10.1186/s12889-015-1929-5)
Supplement: Additional file 1: — Non-HDL target and prevalence of cardiovascular risk factors stratified for age group (40–59, 60–79). [file 12889_2015_1929_MOESM1_ESM.pdf]

Additional File 1

Table 1: Prevalence of Non-HDL above the target (95% confidence interval)

	No CHD or stroke (>3.8 mmol/l) (N=4489)		CHD or stroke (>2.6mmol/l) (N=612)	
	58.8 (56.6-61)		86.3 (82.3-89.5)	
Age	Men (N=2040)	Women (N=2449)	Men (N=396)	Women (N=216)
Total	63	54.9	86.3	86.3
	(60.1-65.9)	(52.1-57.6)	(81.9-89.8)	(79.1-91.3)
40-59 years	62.5	45.4	85.6	92.5
	(59-66)	(42.2-48.7)	(72.2-93.2)	(66.7-98.7)
60-79 years	64.1	70.6	86.5	85.2
	(59.9-68)	(66.6-74.3)	(81.9-90.1)	(77.3-90.7)

CHD: coronary heart disease, HDL: High density lipoprotein cholesterol

Table 2: Prevalence of cardiovascular risk factors (95% confidence interval) among men by age group

Risk factor	40-59 years				60-79 years			
	Total (N=1230)	No CHD or stroke (N=1164)	CHD or Stroke (N=66)	P	Total (N=1206)	No CHD or stroke (N=876)	CHD or Stroke (N=330)	P
Daily smoking	28.4	27.8	39.7	0.100	10.7	10.9	10.1	0.800
	(24.7-32.4)	(23.9-32.0)	(24.8-56.7)		(8.8-13)	(8.8-13.5)	(6.2-16.1)	
<2.5h physical activity/week	21.3	21.2	23.2	0.700	82.3	81.4	84.4	0.400
	(18.7-24.3)	(18.5-24.3)	(14.1-35.9)		(79.1-85.1)	(77.8-84.5)	(77.3-89.6)	
No day physically active/week	28.2	27.1	49	**0.002**	39.1	35.6	47.9	**0.014**
	(25.0-31.7)	(23.9-30.6)	(35-63.1)		(35.1-43.3)	(31-40.3)	(39.5-56.3)	
<2 portions fruit/day	74.3	74.9	60.6	0.100	65.7	64.1	69.6	0.200
	(71.4-77)	(72-77.6)	(43.7-75.2)		(61.7-69.4)	(59.5-68.5)	(62.4-75.9)	
<2 portions vegetables/day	94	94.1	91.2	0.500	93.7	93.2	94.8	0.500
	(91.9-95.5)	(92-95.7)	(75.9-97.2)		(91.6-95.3)	(91-94.9)	(88.6-97.7)	
<1 portions fish/week	65.6	65.8	62.6	0.700	65.3	62.7	72.1	**0.019**
	(62.2-68.9)	(62.3-69.1)	(47.6-75.6)		(61.8-68.7)	(58.5-66.7)	(65.4-78)	
Alcohol intake> 20g	19.5	19.4	20.9	0.800	24.4	25.3	22.2	0.400

	(16.8-22.5)	(16.7-22.5)	(10.7-36.7)		(21.1-28)	(21.6-29.3)	(16.2-29.6)	
Alcohol intake= 0g/day	8.1	7.8	15.2	0.200	7.9	8.4	6.7	0.500
	(6.1-10.7)	(5.8-10.4)	(5.4-35.8)		(6.2-10.1)	(6.1-11.5)	(4-11)	
Overweight (BMI≥25kg/m²)	73.9	73.4	83.3	0.100	83.5	82.2	86.7	0.100
	(70.6-76.9)	(70.1-76.5)	(70.4-91.3)		(80.7-85.9)	(78.9-85.1)	(81.2-90.7)	
Obesity (BMI≥30kg/m²)	25.1	24.5	36	0.100	32.1	28.4	41.5	**0.004**
	(22-28.5)	(21.4-28)	(22.2-52.6)		(28.3-36.4)	(24.4-32.6)	(33.5-49.9)	
BP≥140/90mmHg	21.5	21.7	18.1	0.500	26.4	28.4	21.7	0.100
	(18.7-24.7)	(18.8-24.9)	(9.6-31.7)		(22.9-30.2)	(24.3-32.8)	(16.3-28.2)	
Proportion of unaware[a] among those with BP≥ 140/90 mmHg	40.9	42.7	-	**0.004**	29.6	34.7	12.7	**0.012**
	(33.7-48.6)	(35.5-50.2)	-		(23.7-36.4)	(27.5-42.7)	(5.2-27.8)	
Proportion treated and aware[b] among those with BP≥140/90mmHg	29.6	27.2	86.6	**<0.001**	55	50.5	70.1	**0.047**
	(22.9-37.3)	(20.8-34.6)	(43.9-98.2)		(48.5-61.4)	(43.2-57.9)	(52.5-83.2)	
Elevated total cholesterol (TC≥ 5.0 mmol/l)	66	67.2	43.3	**0.002**	63.8	72.4	42.4	**<0.001**
	(62.1-69.8)	(63.2-71)	(29-58.9)		(59.5-67.8)	(68.1-76.4)	(34.9-50.3)	
Highly elevated TC (≥6.2 mmol/l)	24.5	24.8	17.8	0.300	20.2	23.8	11.2	**0.003**
	(21.5-27.8)	(21.8-28.1)	(8.8-32.7)		(17.1-23.6)	(20.1-28)	(6.7-18.1)	
Proportion unaware[a] among those with TC≥5.0 mmol/l	62.3	63.1	38.6	0.100	48.8	51.8	36	**0.013**
	(58.1-66.3)	(58.6-67.3)	(19-62.7)		(43.9-53.6)	(46.7-56.8)	25.7-47.7	
Proportion treated and aware[b] among those with TC≥5.0 mmol/l	5.3	4.9	16.4	**0.014**	14.8	10.4	33.3	**<0.001**
	(3.7-7.4)	(3.3-7.2)	(6.5-35.6)		(11.8-18.4)	(7.9-13.7)	(23.5-44.9)	

P values less than 0.05 were considered statistical significant (bold).

BMI: body mass index, BP: blood pressure, CHD: coronary heart disease, TC: total cholesterol

[a] defined as reported diagnosis of hypertension or dyslipidaemia

[b] defined as reported diagnosis of hypertension or dyslipidaemia in combination with reported use of antihypertensive or lipid-lowering drugs, respectively

Table 3: Prevalence of cardiovascular risk factors (95% confidence interval) among women by age group

Risk factor	40-59 years				60-79 years			
	Total (N=1230)	No CHD or stroke (N=1164)	CHD or Stroke (N=66)	P	Total (N=1206)	No CHD or stroke (N=876)	CHD or Stroke (N=330)	P
Daily smoking	24.5	23.9	53.5	**0.004**	8.8	8.6	10	0.600
	(21.6-27.7)	(21.1-27)	(31.1-74.6)		(7-11.1)	(6.6-11.1)	(5.6-17.3)	
<2.5h physical activity/week	84.1	83.9	97.4	**0.027**	85.5	85.7	84.4	0.700
	(81.8-86.2)	(81.5-86)	(82.8-99.7)		(82.2-88.3)	(82.2-88.6)	(75.8-90.4)	
No day physically active/week	32.8	32.5	46.6	0.200	41.2	38.7	54.1	**0.005**

	(29.7-36.2)	(29.3-36)	(24.7-69.8)		(37.3-45.3)	(34.5-43.1)	(44.2-63.6)	
<2 portions fruit/day	65.6	65.4	72.8	0.400	55.4	52.8	68.1	**0.002**
	(62.6-68.4)	(62.4-68.3)	(52.8-86.5)		(51.2-59.4)	(48.3-57.3)	(59.5-75.5)	
<2 portions vegetables/day	86.3	86.2	90.4	0.500	88.1	87.6	90.8	0.300
	(83.2-88.8)	(83.2-88.7)	(72.5-97.1)		(85.3-90.5)	(84.5-90.2)	(84.5-94.6)	
<1 portions fish/week	70.6	70.4	81.1	0.200	66.9	65.8	72.7	0.100
	(67.2-73.7)	(66.9-73.6)	(62.2-91.8)		(63.2-70.4)	(61.8-69.5)	(63.6-80.2)	
Alcohol intake> 10g	16.1	16	20.1	0.800	11.8	13.1	5.3	**0.011**
	(13.9-18.6)	(13.8-18.5)	(4.2-59.1)		(9.6-14.5)	(10.5-16.2)	(2.6-10.5)	
Alcohol intake= 0g/day	15.2	15.1	21.7	0.400	20.8	19.3	28.1	**0.045**
	(12.8-18)	(12.6-17.9)	(9.2-43.1)		(17.4-24.6)	(15.7-23.6)	(20.6-37)	
Overweight (BMI≥25kg/m²)	53.1	52.8	68.0	0.200	75.6	73.4	86.4	**0.001**
	(49.8-56.4)	(49.5-56.1)	(44.6-84.8)		(72.5-78.4)	(70-76.6)	(79.8-91.1)	
Obesity (BMI≥30kg/m²)	22.7	22.6	27	0.600	37.6	34.9	51.6	**0.001**
	(20-25.7)	(19.9-25.6)	(12.3-49.4)		(34.2-41.2)	(31.2-38.7)	(42.8-60.3)	
BP≥140/90mmHg	11.4	11.3	14.3	0.600	26.9	28.5	19.1	**0.044**
	(9.5-13.6)	(9.5-13.5)	(4.6-36.8)		(23.6-30.5)	(24.9-32.4)	(12.7-27.7)	
Proportion of unaware[a] among those with	42.7	43	33.4	0.700	22.9	24.2	13.3	0.200
BP≥ 140/90 mmHg	(33.4-52.6)	(33.5-53)	(5.2-82.1)		(17.7-29)	(18.5-30.9)	(4.9-31.2)	
Proportion treated and aware[b] among	29.5	30.3	-	0.200	65.1	63.9	74.4	0.300
those with BP≥140/90mmHg	(22.1-38.2)	(22.7-39.1)	-		(58.9-70.9)	(57.2-70.1)	(54.1-87.8)	
Elevated total cholesterol (TC≥5.0	67.6	67.7	59.1	0.400	79.1	82.9	59.4	**<0.001**
mmol/l)	(64.3-70.6)	(64.5-70.8)	(35.9-78.8)		(75.6-82.1)	(79.4-86)	(50-68.2)	
Highly elevated TC (≥6.2 mmol/l)	21.1	21	24.7	0.800	34.3	37.3	18.8	**<0.001**
	(18.3-24)	(18.3-23.9)	(7.7-56.5)		(30.6-38.1)	(33.3-41.6)	(12.6-27.2)	
Proportion unaware[a] among	66.6	66.9	50.1	0.300	46.9	49.2	30.1	**0.004**
those with TC≥5.0 mmol/l	(62.4-70.6)	(62.7-70.8)	(19.7-80.4)		(43-50.8)	(45.1-53.3)	(20-42.7)	
Proportion treated and aware[b] among	4.3	4.3	4.1	0.900	15.3	13.2	30.3	**<0.001**
those with TC≥5.0 mmol/l	(3-6.3)	(3-6.3)	(0.5-26)		(12.6-18.5)	(10.5-16.5)	(21.3-41.2)	

P values less than 0.05 were considered statistical significant (bold).

BMI: body mass index, BP: blood pressure, CHD: coronary heart disease, TC: total cholesterol

[a] defined as reported diagnosis of hypertension or dyslipidaemia

[b] defined as reported diagnosis of hypertension or dyslipidaemia in combination with reported use of antihypertensive or lipid-lowering drugs, respectively
