# Supplementary material for: Modifiable cardiovascular risk factors in adults aged 40–79 years in Germany with and without prior coronary heart disease or stroke
Source: BMC Public Health. 2015 Jul 24;15:701. doi: 10.1186/s12889-015-1929-5 (PMC4513378; doi:10.1186/s12889-015-1929-5)
Supplement: Additional file 2: — Prevalence of cardiovascular risk factors by obesity status, blood pressure level, and total cholesterol level. [file 12889_2015_1929_MOESM2_ESM.pdf]

Additional File 2

Table 1: Prevalence of cardiovascular risk factors (95% confidence interval) by obesity status

Risk factor	Non-obese (BMI< 30) N=3,658	Obese (BMI≥ 30) N=1,408	P
Daily smoking	20.6 (18.7-22.8)	17.8 (15-21)	0.100
< 2.5 h physical activity/week	82.3 (80.6-83.9)	82.8 (80.1-85.1)	0.700
Physically inactive (no physical activity in average week)	39 (35.4-42.7)	32.6 (30.4-34.9)	**0.002**
< 2 portions fruit/day	65.7 (63.7-67.6)	67.4 (64-70.5)	0.400
< 2 portions vegetable/day	90.5 (89.1-91.7)	90.3 (87.8-92.3)	0.900
< 1 portion fish/week	67.5 (65.4-69.5)	67.3 (63.9-70.5)	0.900
Alcohol intake > 10g/day (women) or 20g/day (men)	19.5 (18-21)	14.1 (11.4-17.3)	**0.003**
Alcohol intake = 0 g/day	10.7 (9.3-12.4)	17.6 (14.7-21)	**<0.001**
BP≥ 140/90 mmHg	17.9 (16.2-19.8)	27.3 (24-30.8)	**<0.001**
Elevated TC (≥ 5.0 mmol/l)	69.3 (66.7-71.8)	67.9 (64.2-71.4)	0.500

P values less than 0.05 were considered statistically significant (bold).
BMI: body mass index, BP: blood pressure, TC: total cholesterol

Table 2: Prevalence of cardiovascular risk factors (95% confidence interval) by blood pressure level

Risk factor	BP< 140/90mmHg N=3,963	BP≥ 140/90mmHg N=1,127	P
Daily smoking	21.2 (19.2-23.3)	14.4 (11.8-17.3)	**<0.001**
< 2.5 h physical activity/week	82.1 (80.6-83.6)	83.5 (80.2-86.3)	0.400
Physically inactive (no physical activity in average week)	39 (35.4-42.7)	32.6 (30.4-34.9)	0.700
< 2 portions fruit/day	65.8 (63.6-67.8)	67.2 (63.7-70.6)	0.500
< 2 portions vegetable/day	90.1 (88.6-91.4)	91.6 (89.2-93.5)	0.300
< 1 portion fish/week	67.6 (65.8-69.5)	66.1 (62.2-69.9)	0.500
Alcohol intake > 10g/day (women) or 20g/day (men)	16.8 (15.3-18.5)	22.2 (19.2-25.6)	**0.002**
Alcohol intake = 0 g/day	12.9 (11.3-14.7)	11.7 (9-15)	0.500
Overweight (BMI ≥ 25 kg/m²)	67 (65-68.9)	81.2 (78.1-83.9)	**<0.001**
Obesity (BMI ≥ 30 kg/m²)	25.9 (24-27.9)	37.5 (33.7-41.5)	**<0.001**
Elevated TC (≥ 5.0 mmol/l)	67.3 (64.8-69.6)	74.4 (70.4-78.1)	**0.001**

P values less than 0.05 were considered statistically significant (bold).
BMI: body mass index, BP: blood pressure, TC: total cholesterol

Table 3: Prevalence of cardiovascular risk factors (95% confidence interval) according by total cholesterol level

**Risk factor**	**TC< 5.0 mmol/l N=1,500**	**TC≥ 5.0 mmol/l N=3,552**	**P**
Daily smoking	19.9 (17.2-22.9)	19.7 (17.8-21.8)	0.900
< 2.5 h physical activity/week	83.5 (81-85.5)	82 (80.2-83.7)	0.300
Physically inactive (no physical activity in average week)	36.2 (32.8-39.8)	33.4 (31.2-35.7)	0.100
< 2 portions fruit/day	65.5 (62.4-68.4)	66.4 (64.2-68.6)	0.600
< 2 portions vegetable/day	90.1 (88.1-91.9)	90.5 (89-91.8)	0.800
< 1 portion fish/week	67.9 (64.4-71.1)	67.1 (64.7-69.3)	0.700
Alcohol intake > 10g/day (women) or 20g/day (men)	16.5 (13.8-19.7)	18.5 (17-20.2)	0.200
Alcohol intake = 0 g/day	12.7 (10.4-15.4)	12.7 (11.2-14.4)	0.900
Overweight (BMI ≥ 25 kg/m²)	67.8 (64.7-70.7)	70.9 (68.9-72.8)	0.100
Obesity (BMI ≥ 30 kg/m²)	29.2 (25.9-32.7)	27.9 (25.9-30)	0.500
BP≥ 140/90 mmHg	16.8 (14.3-19.8)	22.3 (20.4-24.4)	**0.001**

P values less than 0.05 were considered statistically significant (bold).
BMI: body mass index, BP: blood pressure, TC: total cholesterol
